# Modulation of the Cardiovascular Risk in Type 1 Diabetic Rats by Endurance Training in Combination with the Prebiotic Xylooligosaccharide

**DOI:** 10.3390/ijms251810027

**Published:** 2024-09-18

**Authors:** Mariya Choneva, Slavi Delchev, Petar Hrischev, Ivica Dimov, Krasimir Boyanov, Iliyan Dimitrov, Fanka Gerginska, Katerina Georgieva, Mariana Bacelova, Anelia Bivolarska

**Affiliations:** 1 Department of Medical Biochemistry, Faculty of Pharmacy, Medical University of Plovdiv, 4000 Plovdiv, Bulgaria; ivica.dimov@mu-plovdiv.bg (I.D.); krasimir.boyanov@mu-plovdiv.bg (K.B.); iliyan.dimitrov@mu-plovdiv.bg (I.D.); anelia.bivolarska@mu-plovdiv.bg (A.B.); 2 Department of Anatomy, Histology and Embryology, Faculty of Medicine, Medical University of Plovdiv, 4000 Plovdiv, Bulgaria; slavi.delchev@mu-plovdiv.bg (S.D.); fanka.gerginska@mu-plovdiv.bg (F.G.); 3 Department of Physiology, Faculty of Medicine, Medical University of Plovdiv, 4000 Plovdiv, Bulgaria; petar.hrischev@mu-plovdiv.bg (P.H.); katerina.georgieva@mu-plovdiv.bg (K.G.); mariana.batselova@mu-plovdiv.bg (M.B.)

**Keywords:** type 1 diabetes, diabetic cardiomyopathy, xylooligosaccharides, endurance training, mitochondrial function, myocardial fibrosis, glycogen content

## Abstract

Diabetic cardiomyopathy is a major etiological factor in heart failure in diabetic patients, characterized by mitochondrial oxidative metabolism dysfunction, myocardial fibrosis, and marked glycogen elevation. The aim of the present study is to evaluate the effect of endurance training and prebiotic xylooligosaccharide (XOS) on the activity of key oxidative enzymes, myocardial collagen, and glycogen distribution as well as some serum biochemical risk markers in streptozotocin-induced type 1 diabetic rats. Male Wistar rats (n = 36) were divided into four diabetic groups (n = 9): sedentary diabetic rats on a normal diet (SDN), trained diabetic rats on a normal diet (TDN), trained diabetic rats on a normal diet with an XOS supplement (TD-XOS), and sedentary diabetic rats with an XOS supplement (SD-XOS). The results show that aerobic training managed to increase the enzyme activity of respiratory Complex I and II and the lactate dehydrogenase in the cardiomyocytes of the diabetic rats. Furthermore, the combination of exercise and XOS significantly decreased the collagen and glycogen content. No significant effects on blood pressure, heart rate or markers of inflammation were detected. These results demonstrate the beneficial effects of exercise, alone or in combination with XOS, on the cardiac mitochondrial enzymology and histopathology of diabetic rats.

## 1. Introduction

Type 1 diabetes mellitus (T1DM) is an organ-specific autoimmune disease with increasing prevalence worldwide [1]. The disease results from an autoreactive T-cell-mediated destruction of the β-cells of the pancreas [2]. The chronic hyperglycemic environment and the oxidative stress and low-grade inflammation associated with it are drivers of various tissue and organ damages in subjects with diabetes [3].

Cardiovascular disease (CVD) is recognized as a leading cause of morbidity and mortality among diabetic patients [1]. Atherosclerotic vascular complications, arterial hypertension and myocardial infarction are among the most common complications of diabetes [4]. Nevertheless, a major etiological factor for heart failure in patients with diabetes that occurs independently of the vascular complications is diabetic cardiomyopathy (DCM) [5]. The latter is presented with an abnormal structure and performance of the myocardium, manifested by myocardial fibrosis, systolic and diastolic dysfunction [6], and marked glycogen elevation [7]. Furthermore, mitochondrial oxidative metabolism dysfunction has been implicated as a key factor in the pathogenesis of DCM [5].

The gut microbiota is a highly heterogenous collection of microorganisms that exists in a symbiotic relation with the host and exerts multiple essential functions contributing to the host’s health [8]. Increasing evidence implies that the gut microbiota and some of its metabolites such as trimethylamine and short-chain fatty acids (SCFAs) significantly contribute to the pathophysiology of DCM. SCFAs, primarily acetate, propionate and butyrate, are believed to exert a protective effect on the cardiovascular system due to their ability to reduce systemic inflammation and their antioxidant activity [8,9].

Dietary oligosaccharides are non-digestible carbohydrate fibers with prebiotic properties that beneficially affect the composition and metabolism of the intestinal microbiota and thus confer diverse health benefits such as amelioration of diabetes and chronic heart diseases [10,11]. Xylooligosaccharides (XOSs) are a type of prebiotic proven to increase the abundance of *Bifidobacterium* spp. in the colon and stimulate the production of fecal SCFAs [12].

It is known that physical exercise beneficially affects the heart in diabetic subjects and can be used as a non-pharmaceutical approach for influencing the disease and its complications. It is found that moderate-intensity training improves the myocardial structure and cardiac function and leads to favorable cardiovascular adaptations [13]. Furthermore, physical exercise protects the myocardium by improving the metabolism of cardiomyocites, inhibiting apoptosis and ameliorating microvascular disorders; ultimately, it is proposed to have the potential non-pharmacological impact to protect against diabetic cardiomyopathy [14]. 

With CVD being a leading cause of mortality in T1DM [15] and due to the lack of specific treatment of DCM [7], we aimed to evaluate the potential beneficial effect of endurance training and the prebiotic XOS on cardiovascular comorbidities and associated with them risk markers in an experimental model of type 1 diabetes. 

## 2. Results

### 2.1. Somatometric Parameters, Blood Pressure, Heart Rate and Maximum Time to Exhaustion

The results for the somatometric parameters are presented in Table 1. The obtained data indicate that aerobic training and XOS, alone or in combination, did not lead to significant differences in the somatometric parameters—abdominal circumference (AC) and body mass index (BMI). No significant effect was observed on the systolic (SBP) and diastolic blood pressure (DBP), and the heart rate (HR) in between groups. 

Our results show that the applied training had a significant effect on the maximum time to exhaustion (MTE). The rats from the Trained type 1 diabetic rats on a normal diet (TDN) group had higher maximal endurance in comparison with the Sedentary type 1 diabetic rats on a normal diet (SDN) group (790.00 ± 121.14 s vs. 561.11 ± 107.41 s, *p* = 0.001). The Trained type 1 diabetic rats on a normal diet with an XOS supplement (TD-XOS) group also had a higher MTE in comparison with the SDN group (811.11 ± 113.88 s vs. 561.11 ± 107.41 s, *p* = 0.000). We did not find significant differences in the MTE between the TDN and TD-XOS groups (*p* > 0.05). 

### 2.2. Biochemical Parameters

**Figure 1:**
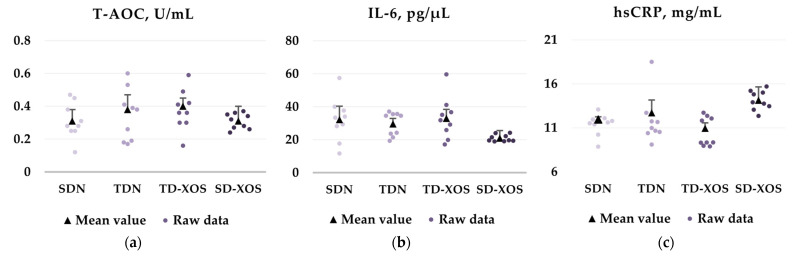
Effects of aerobic training and XOS on the biochemical parameters. (**a**) Serum T-AOC; (**b**) Serum levels of IL-6; (**c**) Serum levels of hsCRP. The graphs present the raw data (n = 9) as well as mean ± SD values. SDN—Sedentary type 1 diabetic rats on a normal diet; TDN—Trained type 1 diabetic rats on a normal diet; TD-XOS—Trained type 1 diabetic rats on a normal diet with an XOS supplement; SD-XOS—Sedentary type 1 diabetic rats on a normal diet with an XOS supplement.

The results for the measured biochemical parameters are presented in Figure 1. The conducted statistical analysis did not detect significant intergroup differences in the serum levels of the studied markers of inflammation—interleukin-6 (IL-6) and high-sensitivity C-reactive protein (hsCRP) and the total antioxidant capacity (T-AOC).

The obtained results suggest that aerobic training conducted for 7 weeks independently or in combination with an XOS supplement failed to reduce the systemic inflammation. The lack of effect of XOS supplementation (group SD-XOS) on the serum levels of IL-6 and hsCRP was reported previously [16]. Neither aerobic training nor XOS exerted a significant effect on the antioxidant defense in the trained and/or supplemented diabetic rats compared to the diabetic control group.

### 2.3. Enzyme Activities of Cardiomyocytes

**Figure 2:**
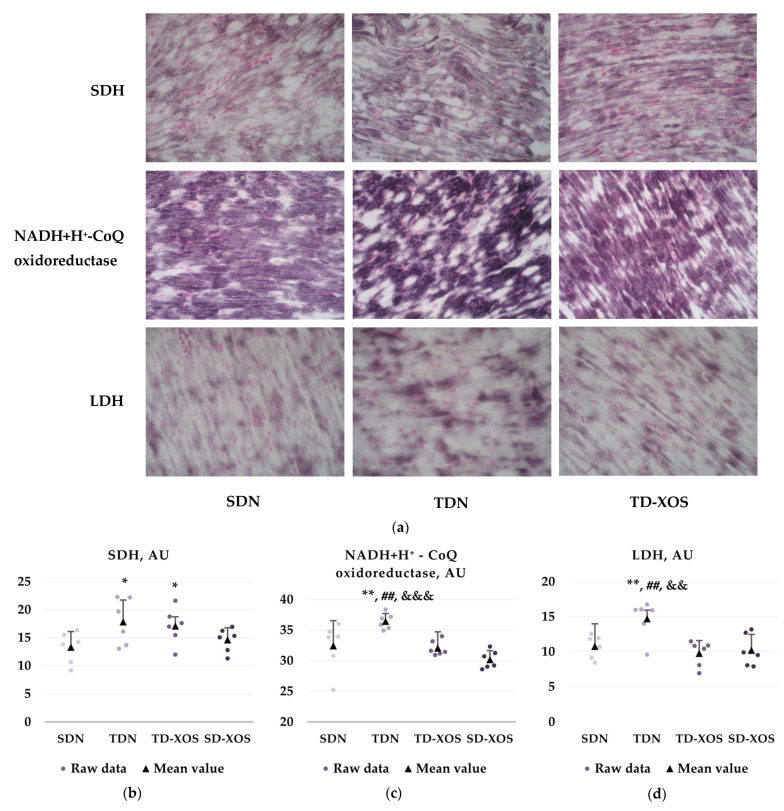
Effects of aerobic training and XOS on the enzyme activity of SDH, NADH + H^+^-CoQ oxidoreductase and LDH in the myocardium of animals from the experimental groups (n = 6). (**a**) Microphotographs of the myocardium used for the enzyme histochemical assessment (magnification ×200). Enzyme activities of SDH (**b**), NADH + H^+^-CoQ oxidoreductase (**c**) and LDH (**d**) (AU) in cardiomyocytes of hearts of the experimental animals at the end of the experiment. The graphs present the raw data as well as mean ± SD values. *—*p* < 0.05 vs. SDN; **—*p* < 0.01 vs. SDN, ##—*p* < 0.01 vs. TD-XOS; &&—*p* < 0.01 vs. SD-XOS; &&&—*p* < 0.001 vs. SD-XOS. SDN—Sedentary type 1 diabetic rats on a normal diet; TDN—Trained type 1 diabetic rats on a normal diet; TD-XOS—Trained type 1 diabetic rats on a normal diet with an XOS supplement; SD-XOS—Sedentary type 1 diabetic rats on a normal diet with an XOS supplement. Note: Photographs of group SD-XOS were not included due to a lack of significant differences from the diabetic control group (SDN).

Figure 2 presents the data from the analysis conducted for assessment of the activity of the marker enzymes: succinate dehydrogenase (SDH, Krebs cycle enzyme and Complex II of the respiratory chain), NADH + H^+^-CoQ oxidoreductase (Complex I of the respiratory chain), and lactate dehydrogenase (LDH) in the cardiomyocytes of the experimental animals. Microphotographs of heart sections used for the analysis are presented in Figure 2a. The data from the enzyme histochemical analysis are presented in Figure 2b–d.

#### 2.3.1. Enzyme Activity of SDH

The analysis of enzyme histochemical reactions (Figure 2b) showed that the trained and prebiotic treated rats had higher cardiomyocyte SDH activity compared to the sedentary diabetic rats (17.07 ± 3.23 vs. 13.29 ± 2.81 arbitrary units (AU), *p* < 0.05). The trained animals on a standard diet also had a higher activity of SDH in the cardiomyocytes compared to the SDN group (17.82 ± 4.13 vs. 13.29 ± 2.81 AU, *p* < 0.05).

#### 2.3.2. Enzyme Activity of NADH + H^+^-CoQ Oxidoreductase

The TDN group had higher NADH + H^+^-CoQ oxidoreductase myocardial activity compared to the sedentary diabetic animals (36.43 ± 1.28 vs. 32.41 ± 3.91 AU; *p* < 0.01), the trained diabetic animals to which XOS was administered (36.43 ± 1.28 vs. 32.04 ± 1.23 AU; *p* < 0.01) and the sedentary diabetic rats supplemented with XOS (36.43 ± 1.28 vs. 30.17 ± 1.47 AU; *p* < 0.001) (Figure 2c).

#### 2.3.3. Enzyme Activity of LDH

The observed changes in the enzyme activity of LDH (Figure 2d) were similar to the ones detected for NADH + H^+^-CoQ oxidoreductase. The trained rats on a normal diet presented with greater activity of the enzyme compared to the sedentary diabetic rats (14.71 ± 2.67 vs. 10.76 ± 1.66 AU; *p* < 0.01), as well as the trained diabetic rats supplemented with XOS (14.71 ± 2.67 vs. 9.77 ± 1.82 AU; *p* < 0.001) and the sedentary diabetic rats supplemented with XOS (14.71 ± 2.67 vs. 10.21 ± 2.26 AU; *p* < 0.01).

The obtained data suggest that aerobic training alone or in combination with XOS was able to increase the enzymatic activity of SDH, while the activities of NADH + H^+^-CoQ oxidoreductase and LDH were only enhanced by the independent effect of exercise. Administration of XOS to the sedentary diabetic rats failed to beneficially affect the studied enzyme activities. 

### 2.4. Collagen and Glycogen Content

**Figure 3:**
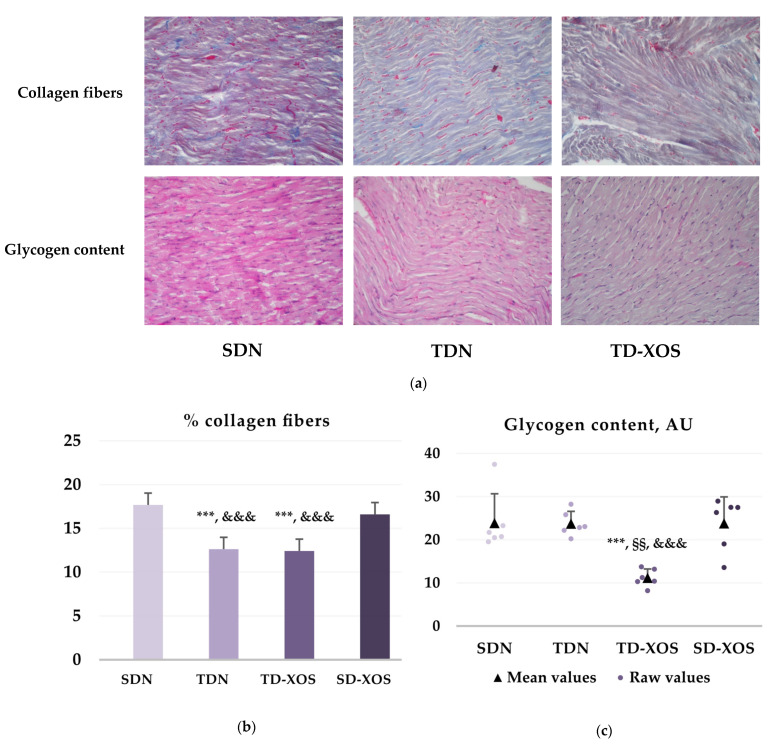
Effects of endurance training and XOS on the distribution of collagen fibers and glycogen in the myocardium of animals from the experimental groups (n = 6). (**a**) Upper row: Collagen fibers (in blue), AZAN staining. Bottom row: Glycogen content, PAS reaction (magnification ×200). (**b**) Percentage of collagen fiber distribution and (**c**) glycogen content, AU (raw data and mean ± SD values) in the cardiomyocytes of the LV of the heart. ***—*p* < 0.001 vs. SDN, §§—*p* < 0.01 vs. TDN, &&&—*p* < 0.001 vs. SD-XOS. SDN—Sedentary type 1 diabetic rats on a normal diet; TDN—Trained type 1 diabetic rats on a normal diet; TD-XOS—Trained type 1 diabetic rats on a normal diet with an XOS supplement; SD-XOS—Sedentary type 1 diabetic rats on a normal diet with an XOS supplement. Note: Photographs of group SD-XOS were not included due to a lack of significant differences from the diabetic control group (SDN).

Results from the evaluation of the effect of endurance training and XOS on the collagen and glycogen content are presented in Figure 3. Microphotographs of the AZAN-stained and the Periodic acid-Schiff (PAS)-stained sections of the left ventricle (LV) of the heart of the experimental animals are presented in Figure 3a. 

The conducted analysis on the percentage of collagen fibers (Figure 3b) discovered a reduction in collagen distribution in the LV of the animals from both trained groups in comparison to the sedentary group (12.63 ± 1.41 vs. 17.68 ± 2.81, *p* < 0.001; 12.42 ± 0.91 vs. 17.68 ± 2.81, *p* < 0.001). XOS supplementation did not additionally affect the amount of collagen (TDN vs. TD-XOS, *p* > 0.05). Furthermore, both trained groups had a lower percentage of collagen fibers compared to the sedentary rats supplemented with XOS (12.63 ± 1.41 vs. 16.6 ± 1.15, *p* < 0.001; 12.42 ± 0.91 vs. 16.6 ± 1.15, *p* < 0.001). 

The results for glycogen content in the LV of the rats are presented in Figure 3c. No significant effect of endurance exercise on the accumulation of glycogen in the myocardium versus the SDN group was found (23.73 ± 2.84 vs. 23.86 ± 6.79 AU; *p* > 0.05). The application of XOS to the trained animals reduced the amount of glycogen in the cardiomyocytes compared to the sole effect of training (11.18 ± 2.04 vs. 23.73 ± 2.84 AU; *p* < 0.001). Furthermore, the combined effect of training and a prebiotic significantly decreased the glycogen contents in the myocardium in comparison to the sedentary diabetic group on a standard diet (11.18 ± 2.04 vs. 23.86 ± 6.79 AU; *p* < 0.001). No significant difference was observed between the prebiotic supplemented sedentary group and the SDN group (23.80 ± 6.13 vs. 23.86 ± 6.79 AU; *p* > 0.05). 

In summary, according to the obtained results, the combination of endurance exercise and XOS managed to reduce the content of both collagen and glycogen in the myocardium of diabetic rats, while endurance training conducted independently only affected the percentage of collagen fiber distribution. Interestingly, the supplementation of the sedentary diabetic rats with XOS failed to affect the distribution of collagen and glycogen in comparison to the control group. 

## 3. Discussion

The present study evaluated the effects of aerobic training and XOS, alone or in combination, on myocardial enzymology, collagen, and glycogen distribution in the LV of type 1 diabetic rats. We further investigated the changes in the systolic and diastolic blood pressure, heart rate, and maximum time to exhaustion as well as changes in the levels of serum proinflammatory markers and T-AOC. Our data showed that the administration of streptozotocin (STZ) leads to impaired mitochondrial bioenergetics, interstitial fibrosis, and increased glycogen abundance in the LV of the experimental animals.

The heart is an organ with a high energy demand [17]. With a reduced insulin-dependent uptake of glucose, the diabetic heart relies almost exclusively on fatty acid oxidation (FAO) for adenosine triphosphate synthesis. This metabolic shift is known as metabolic inflexibility [18]. In addition to these metabolic changes, the cardiac dysfunction in diabetes is believed to result from defective mitochondrial bioenergetics [19]. Several studies have reported severe impairments in respiratory Complex I and II in diabetes [19,20]. 

Our experimental model demonstrated decreased SDH and NADH + H^+^-CoQ oxidoreductase activity of the diabetic control group in comparison to the trained animals. Aerobic training, alone or in combination with XOS, improved the oxidative phosphorylation (OXPHOS) in complex II of the respiratory chain, while increased Complex I activity was only observed as a result of exercise. These data are consistent with the findings of other authors [21,22]. The beneficial effect of endurance training on OXPHOS is suggested to result from induced upregulation of the proliferator-activated receptor gamma coactivator 1 alpha—a transcription coactivator that increases the expression of electron transport chain proteins [21]. 

According to our results, XOS supplementation failed to increase the enzyme activity of respiratory complex I and II and furthermore appeared to diminish the beneficial effect of endurance training on the activity of NADH + H^+^-CoQ oxidoreductase. A possible explanation for the discrepancy could be inhibition of glycolysis and FAO of long-chain fatty acids—a reported effect of the oligosaccharide [23]. Other authors have reported that XOS supplementation has been shown to increase the protein expression of respiratory Complex I [11]; however, their treatment regimen included a dose twice as high as the one administered to our experimental animals. 

Apart from the impaired mitochondrial OXPHOS, the metabolism of the diabetic heart is additionally characterized by a significant decrease in lactate oxidation [24,25]. In our study, the total LDH activity in the presence of lactate as a substrate was increased by aerobic training, while XOS supplementation as well as the combination of training and XOS had no influence. Exercise has been reported to markedly elevate the utilization of lactate as an energy source in the heart [26,27]. It has been proven that continuous production of lactate could take place under aerobic conditions, which is important for the provision of energy sources to the heart [28]. The increased LDH activity and oxidation of lactate could supplant cardiac FAO and thus ameliorate the metabolic inflexibility in the diabetic state [29]. The lack of effect of XOS and the combination of XOS and exercise on LDH activity could be explained by the increased levels of butyrate—a short chain fatty acid, produced by the colon upon fiber fermentation, that is reported to reduce the expression of the enzyme in rats [30,31]. 

Cardiac fibrosis is characterized by elevated interstitial collagen distribution that results from an unbalanced metabolism of the extracellular matrix, expressed by an increased rate of collagen synthesis and decreased rate of its degradation [13]. The reduced turnover of collagen in diabetes is associated with chronic hyperglycemia that induces the spontaneous glycation of proteins leading to accumulation of advanced glycation end-products (AGEs) [32]. Collagen glycation is reported to induce the formation and subsequent migration of myofibroblasts, which is considered a key event in diabetic fibrosis development [33].

Our results demonstrate increased collagen deposition in the cardiomyocytes of STZ-induced type 1 diabetic rats. Furthermore, interstitial fibrosis was significantly more evident in the diabetic control group in comparison to both trained groups with and without the addition of a prebiotic. The observed positive effects of treadmill exercise on the interstitial fibrosis of the experimental animals confirm the ones reported in the literature [34,35]. Multiple mechanisms have been proposed through which exercise training attenuates LV fibrosis. One of them is the inhibition of the transforming growth factor-β1/Smad signaling pathway, which is associated with increased expression of the tissue inhibitor of metalloproteinase protein and a decreased rate of collagen degradation [34]. Furthermore, exercise is found to stimulate mitochondrial adaptations [36] and to reduce oxidative stress [37] and the formation of AGEs, all of which related to myocardial fibrosis development [34]. 

As is evident from the results, no significant independent effect was observed for the xylooligosaccharide. A beneficial effect of XOS on myocardial fibrosis in mice with heart failure was reported, but with the oligosaccharide only being a component of a decoction [38]. Another type of oligosaccharide has been found to attenuate cardiac fibrosis in rats [39]; however, at doses much higher than the one we used.

While interstitial fibrosis is generally common in HF, the accumulation of glycogen is considered an essential feature underlying DCM [40]. The redirection of glucose metabolism towards glycogen synthesis is suggested to result from the decreased glucose transport and augmented fatty acid metabolism accompanied by suppressed glucose catabolism [7,13]. As is evident from our results, there was a marked glycogen elevation in the diabetic myocardium of the control group. These data confirm the results obtained by Mellor et al. [7]. Aerobic training conducted for 7 weeks did not significantly affect the cardiac glycogen content of the diabetic rats. These findings are consistent with the ones reported by Silva et al. [13]. The supplementation of the sedentary diabetic rats with XOS did not reduce the glycogen content. The reported effects of oligosaccharides on cardiac glycogen metabolism are contradictory. Sarfaraz et al. discovered that oligosaccharide treatment increased the level of the enzyme glycogen phosphorylase, while simultaneously decreasing the ones of phosphoglucomutase [41]. Interestingly, in our study the addition of an XOS supplement to the exercise regimen resulted in a notable decrease in the amount of glycogen. To our knowledge, this is the first study to investigate the effect of XOS in combination with endurance training on the distribution of glycogen in the myocardium of STZ-induced type 1 diabetic rats. Further studies will be needed for the mechanism of action of exercise and XOS to be fully understood. 

Hypertension is often observed in diabetic patients as both conditions share risk factors. The concomitant existence of these morbidities is associated with an elevated risk of CVD. Hence, the control of hypertension through the usage of medication and proper lifestyle changes is critical if complications are to be avoided [42]. According to our results, the applied aerobic training and oligosaccharide did not lead to changes in the systolic and diastolic blood pressure and the heart rate compared to the diabetic controls. Similar to our findings, Wróbel et al. did not observe significant differences in the blood pressure and heart rate of the control versus exercised group [43]. 

Our results showed that aerobic training conducted for 7 weeks improves the maximal endurance of the exercised rats. These findings confirm the results of Souza et al. [44], who reported that female trained animals with streptozotocin-induced type 1 diabetes have better endurance in comparison with the non-trained. In contrast to our results, Smirnova et al. [45] did not find significant differences between trained and non-trained 7-month-old rats with T1DM. We found no effect of the oligosaccharide used concomitantly with the exercise training on the working capacity of the diabetic rats.

The development of DCM is intricate and based on impairments in glucose metabolism, oxidative stress damage, and the activation of various inflammatory pathways [38]. The total antioxidant capacity evaluates the cumulative effect of all antioxidants present in the serum [46]. According to our results, aerobic training and XOS supplementation had no effect on the T-AOC of the experimental animals compared to the diabetic controls. A significant increase in the T-AOC in an animal model of type 1 diabetes has been reported by Ghyasi et al. [47]. The discrepancy in the obtained data could be explained by the different model of the training, that includes voluntary treadmill exercise. The data regarding the effect of oligosaccharides on serum T-AOC conflict. A significant increase in the marker post prebiotic treatment was reported by Gao et al. [48], while Żary-Sikorska and Juśkiewicz [49] found no effect.

The obtained results on the serum levels of IL-6 and hsCRP demonstrate no effect of aerobic training and XOS compared to the diabetic controls. Similar data on the effect of aerobic training on the serum inflammatory markers have been reported by Farinha et al. [50] and Codella et al. [51]. Likely reasons for the lack of effect of aerobic exercise could be insufficient duration or intensity of the training as it has been suggested that the combination of aerobic and resistance training exerts a more pronounced effect on the levels of proinflammatory markers than aerobic exercise alone [52]. The literature data on the effects of oligosaccharides on the serum levels of IL-6 and hsCRP are disputable, with a lack of effect reported by Malardé et al. [53] and Liu et al. [54], and positive effects reported by Morel et al. [55] and Lim et al. [56]. 

Based on the findings on the beneficial effect of endurance training and XOS on the major hallmarks of DCM, potential non-pharmacological therapeutic strategies could be proposed for the protection of the heart in diabetes. Implementation of exercise and an XOS supplement or a high-fiber diet is easily achievable, universally available, and cost-effective. However, due to the knowledge gap, further studies on the exact mechanisms of action, the optimal duration of training and prebiotic supplementation as well as oligosaccharide dosage would be needed before their implementation into clinical practice. 

The limitations of the present study are the lack of a healthy (non-diabetic) control group as well as the lack of assessment of markers of inflammation and oxidative stress in the cardiac tissue. 

## 4. Materials and Methods

### 4.1. Animals and Experimental Design

All experimental protocols were conducted according to the guidelines of the Declaration of Helsinki and the Bulgarian Agency for Food Safety (BAFS resolution No. 150/09.04.2019) and are in compliance with the ethical standards of the Medical University of Plovdiv (resolution of the University Ethics Committee No. 2/13.06.2019).

**Figure 4:**
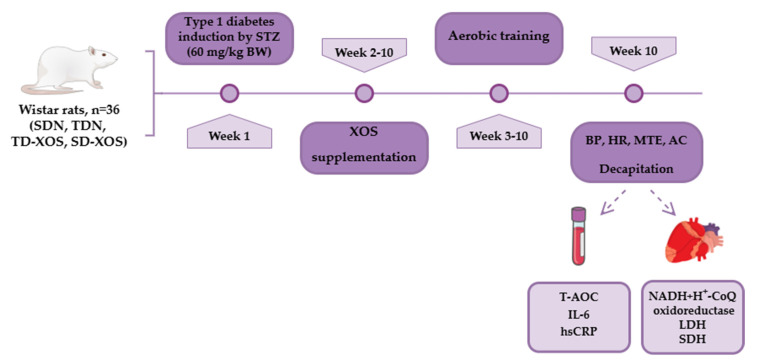
Study design. BP—blood pressure, HR—heart rate, MTE—maximum time to exhaustion, AC—abdominal circumference, T-AOC—total antioxidant capacity, Il-6—interleukin-6, hsCRP—high-sensitive C-reactive protein, NADH + H+—nicotinamide adenine dinucleotide hydride, CoQ—coenzyme Q, LDH—lactate dehydrogenase, SDH—succinate dehydrogenase.

The objects of the experiment were 36 8-week-old male Wistar rats, weighing approximately 195 ± 30 g. The animals were sourced from the vivarium of the Medical University of Plovdiv and were housed in polypropylene cages (four to five in each cage) under standard laboratory conditions (12-h light/dark cycle, living space of 350 cm^2^, relative humidity of 55 ± 10% and temperature of 25 ± 2 °C). The design of the experiment is presented in Figure 4. After a few days of acclimatization period with basal feed and tap water ad libitum, the rats were randomly divided into four groups (n = 9):

Group 1—Sedentary type 1 diabetic rats on a normal diet (SDN).
Group 2—Trained type 1 diabetic rats on a normal diet (TDN).
Group 3—Trained type 1 diabetic rats on a normal diet with an XOS supplement at a dose of 100 mg/kg body weight (BW)/day (TD-XOS).
Group 4—Sedentary type 1 diabetic rats on a normal diet with an XOS supplement at a dose of 100 mg/kg body weight (BW)/day (SD-XOS).

Type 1 diabetes was induced by a single dose (60 mg/kg BW) of the antineoplastic antibiotic STZ dissolved in a citrate buffer and administered intraperitoneally. The STZ dose was chosen based on the report of Akbarzadeh et al. [57]. The rats did not receive antihyperglycemic treatment. The successful induction of a type 1 diabetes-like syndrome was confirmed by a weekly measurement of the blood glucose concentration with the help of a glucometer. The data for the serum glucose level were reported previously [58]. Animals with a blood glucose concentration of 12 mmol/L or above were considered diabetic. 

The rats were kept under standard conditions for 10 weeks. At the end of the experimental period, the animals were fasted for 24 h, treated with an overdose of the anesthetic ketamine/xylazine (87.5/12.5 mg/kg BW), and then decapitated. The blood was collected and centrifuged at 3000 rpm for 5 min. The obtained serum was frozen at −80 °C until the biochemical analysis. The hearts of all experimental animals were dissected and small parts (approx. 5/5/5 mm) of the left ventricle were immediately dipped in liquid nitrogen. Samples were transferred and stored in a refrigerator at −80 °C until the analysis.

### 4.2. Chemicals

STZ (Streptozotocin Mixed Anomers, Sigma-Aldrich, Burlington, MA, USA) was dissolved in a citrate buffer, pH 4.5 in accordance with the method of B. Furman [59]. The buffer was prepared with 5.78 g citric acid × 1 H_2_O (M = 210.14 g/mol) and 0.71 g Na_2_HPO_4_ (M = 141.96 g/mol), each dissolved in 50 mL of distilled water. Ten ml of the citric acid solution and 45–50 mL of the Na_2_HPO_4_ solution were used for the pH of the buffer to be reached. One gram of the dry STZ substance was dissolved in 33.3 mL of buffer recalculated according to the starting dose of 60 mg/kg BW [57].

XOS (Xylooligosaccharide powder, Lenzing AG, Lenzing, Austria) was dissolved in distilled water and orally administered to the rats of the TD-XOS and SD-XOS groups at a dose of 100 mg/kg BW every day for 8 weeks. The degree of polymerization (DP—number of monomers in the chains) of the XOS used is as follows: DP—2 (13%); DP—3 (19%); DP—4 (11%) and DP—5 or more (60%).

### 4.3. Aerobic Training Protocol

Treadmill running is a skill that experimental animals need to develop. Therefore, the rats were trained on a treadmill for small experimental animals, EXER-3R-Treadmill (Columbus Instruments, Columbus, OH, USA), for 5 min/day, 3 days per week before the experiment had begun. This duration of exercise does not lead to adaptive changes [60]; however, it allows for the animals to get used to treadmill running and facilitates the selection of the ones who run spontaneously. The animals from the TDN and TD-XOS groups were subjected to endurance training on a treadmill with a band speed of 16 m/min, a slope of 5°, five days a week for 7 weeks, starting from the third week of the experiment. The exercise duration was 20 min on the first day and was gradually increased by 5 min every other day. At the end of the second week, the duration reached 40 min and it remained unchanged until the end of the experiment. For the functional tests to be performed and the motor skills of the rats to be maintained, the animals from the sedentary group also ran on the treadmill for 5 min 3 days a week at the same speed and band slope. 

### 4.4. Somatometric Parameters

At the end of the experimental period, the AC of the rats was measured at the largest zone of the rat abdomen via a plastic measuring tape. Based on previously reported data on the body weight and naso-anal length of the experimental animals [58], the BMI was calculated as the ratio BW (g)/naso-anal length^2^ (cm^2^) [61].

### 4.5. Blood Pressure and Basal Heart Rate 

The arterial blood pressure (ABP) of the rats was evaluated with a tail-cuff method using a system for non-invasive measurement (NIBP 200A, Biopac Systems Inc., Goleta, CA, USA) and MP150 software version 4.0 for data processing and interpretation (Biopac Systems Inc., Goleta, CA, USA). The measurements were performed on conscious rats at least 2 days after the last physical exercise. A cuff was placed in the widest part of the tail, and the ABP as well as the HR of the animals was registered. For the animals to become accustomed to the new conditions and the stress levels during the measurement to be reduced, prior to the procedure all rats were put in restrainers for 5 min, 3 times per week for 1 week. The SBP and DBP, and the HR values of each rat were taken seven times. To avoid the effects of stress during the first measurement and for stable pressure values to be established, the average value of the last three measurements were used as representative. 

### 4.6. Maximum Time to Exhaustion

At the end of the experiment, three of the groups (SDN, TDN, and TD-XOS) were tested for MTE. The peak load was achieved by a stepwise increase in the speed and inclination of the treadmill band according to protocol—Ist step: 15 m/min, 5°; IInd step: 19 m/min, 10°; IIIrd step: 27 m/min, 10°; IVth step: 27 m/min, 15°; Vth step: 30 m/min, 15°; VIth step: 35 m/min, 15°; VIIth step: 40 m/min, 15°. Each step lasted 3 min [62]. When the rat could no longer hold its position on the treadmill band, the test was terminated. The time to reach this state was assumed to be the MTE.

### 4.7. Serum Biochemical Parameters

Serum T-AOC, IL-6 and hsCRP were analyzed on an enzyme-linked immunosorbent assay (ELISA) microplate reader HumanReader.HS, HUMAN (Wiesbaden, Germany) via commercially available kits [Rat Total antioxidant capacity, T-AOC Elisa kit, Nanjing Pars Biochem CO., Ltd., Nanjing, China; Rat IL-6 (Interleukin 6) ELISA Kit, Elabscience Biotechnology Inc., Houston, TX, USA; Rat hsCRP (high-sensitivity, C-Reactive Protein) Elabscience Biotechnology Inc., Houston, TX, USA].

### 4.8. Enzyme Histochemistry

Myocardium from the middle third of the LV of the heart was excised, immediately deep-frozen in liquid nitrogen and then stored at −80 °C until the conduction of the analysis. Ten μm thick cryostat sections were used for the enzyme histochemical assessment. The sections were prepared on a “Leica CM 1500” cryostat (Leica Instruments, Wetzlar, Germany). Sections parallel to the fibers of the cardiomyocytes were selected for the purposes of the study. We tracked the activity of key marker enzymes: mitochondrial respiration enzymes—SDH (Krebs cycle enzyme and Complex II of the respiratory chain) and NADH + H^+^-CoQ oxidoreductase (Complex I of the respiratory chain) as well as the glycolytic enzyme LDH.

#### 4.8.1. Succinate Dehydrogenase Activity

Sodium succinate was used as the substrate for the reaction. The dried fresh cryostat sections were placed in cuvettes with incubation solution containing polyvinylpyrrolidone (PVP)-0.05, 0.05 M Tris buffer, distilled water, nitrotetrazolium blue chloride (NBT)-0.006, and 0.2 M sodium succinate. The cuvettes were placed in a thermostat at 37 °C for 30 min. This was followed by staining of the nuclei using the histochemical reaction of Feulgren and Rossenbeck [63] with a Schiff reagent, and finally embedding in Canada balsam.

#### 4.8.2. NADH + H^+^-CoQ Oxidoreductase Activity

Nicotinamide adenine dinucleotide reduced disodium salt (DPNH_2_-Na_2_) was used as the substrate. The sections were incubated at 37 °C for 10 min. The incubation solution contained PVP-0.05, Tris buffer (pH 8.05)—0.05 M, MgCl_2_—0.05 M, 0.007 NBT in dimethylformamide, 0.1 M KCN, and DPNH_2_-Na_2_. The nuclei were stained by the Feulgen reaction with the Schiff reagent. Finally, the sections were embedded in Canada balsam.

#### 4.8.3. Lactate Dehydrogenase Activity

Sodium lactate was used as the substrate. The cuvettes with the incubation solution (1M sodium lactate, 0.1 M KCN, PVP 1.0500, 0.05 M Tris buffer, distilled water, 0.05 M MgCl_2_, 0.007 NBT in dimethylformamide, DPN 0.0063) were placed in a thermostat at 37 °C for 20 min. The nuclei were stained by the Feulgen reaction with the Schiff reagent.

The intensity of the reactions for the three studied enzymes was analyzed. With the help of “DP-Soft”software version 3.2 (Olympus, Japan) the average saturation (intensity) of the enzyme histochemical reaction in AU was recorded in the cardiomyocytes of the myocardium from six animals from each experimental group.

### 4.9. Cardiomyocyte Glycogen Content Assessment

For the measurement of the glycogen content, the middle third of the outer wall of the LV was excised and immediately frozen in liquid nitrogen then stored at −80 °C until the conduction of the analysis. For the assessment of glycogen distribution, sections parallel to the long axis of the myocardial muscle fibers were selected. The PAS reaction by McManus was applied. Through this conventional histochemical method and the use of image processing software, the glycogen content is semi-quantitatively assessed in muscle sections [64]. The average saturation (intensity) of the reaction in the cardiomyocytes of six animals from each experimental group was recorded using “DP-Soft” 3.2 (Olympus, Japan) software.

### 4.10. Cardiomyocyte Collagen Content Assessment

A small section of the LV wall of the heart of the rats was fixed in Bouin’s solution for 24 h and embedded in paraffin. Five μm thick paraffin sections were then deparaffinized, rehydrated, and stained with AZAN by Heidenhain. The percentage distribution of connective tissue in the extracellular matrix of longitudinal sections of the myocardium of six rats from each group was recorded. With the help of image analysis software (“DP-Soft” 3.2, Olympus, Japan) and a measuring grid (19 × 25 fields) at 200× magnification, the relative percentage distribution of collagen fibers in the myocardium was calculated using the formula x = (n/475) × 100, where n is the number of squares containing collagen fibers and 475 is the total number of squares.

### 4.11. Statistical Analysis 

The statistical analysis was performed with SPSS Statistics software version 17.0 (SPSS Inc., Chicago, IL, USA). Results are expressed as mean ± standard deviation (SD). Statistical differences between groups were evaluated by one-way analysis of variance (ANOVA) followed by the LSD post hoc test. The statistical significance was set at *p* ≤ 0.05.

## 5. Conclusions

The present experiment demonstrates that endurance training, alone or in combination with an XOS supplementation, exerts significant beneficial effects on key myocardial oxidative enzymes, interstitial fibrosis, and glycogen content in rats with STZ-induced type 1 diabetes. The applied exercise managed to increase the rate of lactate oxidation and OXPHOS, while the addition of XOS favorably affected myocardial collagen and glycogen distribution. XOS supplementation to the sedentary diabetic animals failed to affect the above listed parameters. No significant effects of exercise or XOS were observed for the blood pressure, heart rate, or the serum biochemical markers. Although some findings indicate a potential beneficial effect of endurance exercise and the prebiotic XOS in ameliorating some aspects of DCM, further research will be required for the underlying mechanisms to be understood. 

## Figures and Tables

**Table 1:** Effects of aerobic training and XOS on somatometric parameters, SBP, DBP, HR and MTE.

Parameter	SDN		TDN		TD-XOS		SD-XOS	
	Mean	SD	Mean	SD	Mean	SD	Mean	SD
Abdominal circumference, cm	16.57	0.98	16.63	1.75	18.23	0.79	15.70	1.29
BMI, g/cm^2^	0.54	0.06	0.62	0.16	0.55	0.04	0.59	0.09
Systolic blood pressure, mmHg	113.04	16.89	132.96	13.98	125.30	11.27	-	
Diastolic blood pressure, mmHg	76.04	12.62	91.81	14.16	83.33	10.77	-	
Heart rate, bpm	182.09	24.98	206.78	43.26	217.93	37.78	-	
MTE, s	561.11	107.41	790.00 **	121.14	811.11 **	113.88	-	

Data are presented as mean ± SD (n = 9). The symbol ** indicates significant differences in comparison to the SDN group, *p* < 0.01. SDN—Sedentary type 1 diabetic rats on a normal diet; TDN—Trained type 1 diabetic rats on a normal diet; TD-XOS—Trained type 1 diabetic rats on a normal diet with an XOS supplement; SD-XOS—Sedentary type 1 diabetic rats on a normal diet with an XOS supplement. Note: The symbol “-” indicates missing data. Measurement of SBP, DBP, HR and MTE for the SD-XOS group was not initially included in the experiment; hence, we report on the effect of aerobic training, alone or in combination with XOS.

## Data Availability

The raw data supporting the conclusions of this article will be made available by the authors on request.
